# Datasets of RT spoofing attacks on MIL-STD-1553 communication traffic

**DOI:** 10.1016/j.dib.2019.103863

**Published:** 2019-03-20

**Authors:** Ran Yahalom, David Barishev, Alon Steren, Yonatan Nameri, Maxim Roytman, Angel Porgador, Yuval Elovici

**Affiliations:** a Department of Software and Information System Engineering, Ben-Gurion University of the Negev, Be'er Sheva 84105, P.O. Box 653, Israel; b Cyber@Ben-Gurion University of the Negev (CBG) & Telekom Innovation Labs, Be'er Sheva 84105, P.O. Box 653, Israel; c The Shraga Segal Department of Microbiology, Immunology and Genetics, Faculty of Health Sciences, Ben-Gurion University of the Negev, Be'er Sheva 84105, P.O. Box 653, Israel

## Abstract

The datasets in this article are produced to evaluate the ability of MIL-STD-1553 intrusion detection systems to detect attacks that emulate normal non-periodical messages, at differing attack occurrence rates. And different data representations. We present three streams of simulated MIL-STD-1553 traffic containing both normal and attack messages corresponding to packets that were injected into the bus by a malicious remote terminal. The implemented attacks emulate normal non-periodical communication so detecting them with a low false positive rate is non-trivial. Each stream is separated into a training set of normal messages and a test set of both normal and attack messages. The test sets differ by the occurrence rate of attack messages (0.01%, 0.10%, and 1.00%). Each stream is also preprocessed into a dataset of message sequences so that it can be used for sequential anomaly detection analysis. The sequential test sets differ by the occurrence rate of attack sequences (0.14%, 1.26%, and 11.01%). All dataset files can be found in Mendeley Data, doi:10.17632/jvgdrmjvs3.3.

**Table:** Specifications table

Subject area	*Cyber Security*
More specific subject area	*Anomaly-based network intrusion detection.*
Type of data	*(1) Simulated stream of MIL-STD-1553 communication messages (2) Sequences of simulated MIL-STD-1553 messages derived from a simulated stream.*
How data was acquired	*Streams of MIL-STD-1553 communication messages were generated according to the MIL-STD-1553 standard from packets recorded on a MIL-STD-1553 bus simulator, corresponding to a basic aircraft bus architecture. Sequences were then derived from each stream by splitting it into segments of ten consecutive messages.*
Data format	*Both dataset types are in comma separated values (CSV) files, as specified below.*
Experimental factors	*The bus architecture was set up to include a single malicious remote terminal that injects packets in order to carry out an attack on the aircraft.*
Experimental features	*Each stream corresponds to about a minute of recording with a different amount of conducted attacks in order to generate message stream files with differing attack occurrence ratios.*
Data source location	
Data accessibility	*All datasets are available in Mendeley Data at*https://doi.org/10.17632/jvgdrmjvs3.3[1]
Related research article	R. Yahalom, A. Steren, Y. Nameri, M. Roytman, A. Porgador, and Y. Elovici, “Improving the effectiveness of intrusion detection systems for hierarchical data,” *Knowledge-Based Syst.*, Jan. 2019 [2].

**Table:** 

**Value of the data** • These datasets can help advance research on detection of cyber-attacks on the MIL-STD-1553 protocol which is currently hindered due to lack of available data (mostly due to confidentiality of recordings from buses on operational systems). • The datasets can be used to evaluate and compare two types of IDSs: (1) IDSs that operate on independent/unordered messages from the stream datasets, e.g. [3] and; (2) IDSs that operate on sequential data from the sequence datasets, e.g. [4]. • These datasets can be used to evaluate the ability of a MIL-STD-1553 intrusion detection system (IDS) to detect RT spoofing attacks that are camouflaged as normal non-periodic messages. • The sequence datasets can be used to take advantage of the temporal message ordering in order to detect the RT spoofing attacks as position and/or combination anomalies [5]. • The datasets can be used to analyze the effect of differing attack occurrence rates on the evaluated IDS.

## 1 Data

**Fig. 1:**
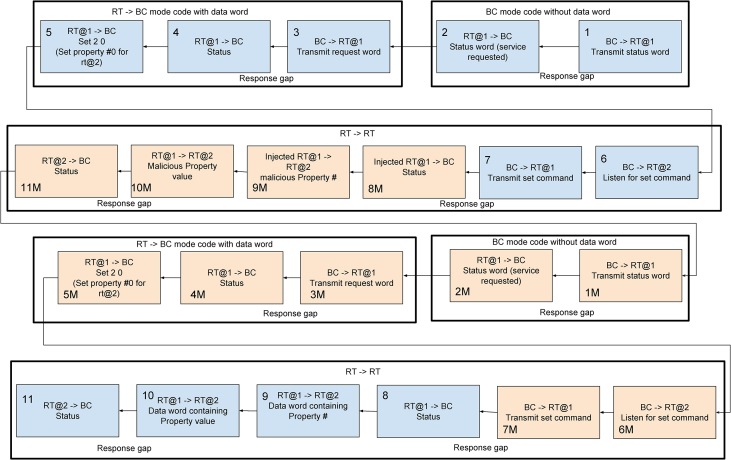
RT spoofing attack via set request.

**Fig. 2:**
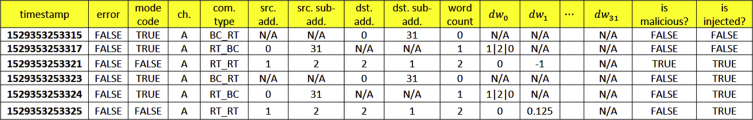
Format of simulated MIL-STD-1553 messages.

**Fig. 3:**
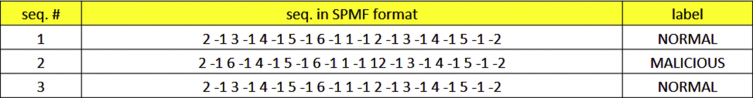
Format of the CSV files storing the derived MIL-STD-1553 sequence datasets, showing three example sequences. The sequences of message identifiers are in SPMF [8] format.

The presented datasets [1] include three non-sequential datasets and three sequential datasets, where each dataset type corresponds to a different CSV file format. The non-sequential dataset files represent a stream of messages generated according to the MIL-STD-1553 standard [6] from packets recorded on a MIL-STD-1553 bus simulator. The bus simulator corresponds to a basic aircraft bus architecture and Fig. 1 explains how a malicious remote terminal (RT) injects packets into this bus in order to carry out a single attack that causes the aircraft to tilt sideways. The three non-sequential datasets were generated by recording about a minute of bus traffic while conducting a different number of repetitions of the same attack. As a result the three test sets differ by the occurrence rate of attack messages (0.01%, 0.10%, and 1.00%). Fig. 2 describes the format of the non-sequential dataset files. Each sequential dataset file includes sequences derived one of the non-sequential dataset files by splitting the message stream into segments of ten consecutive messages. Fig. 3 describes the format of the sequential dataset files.

## 2 Experimental design, materials, and methods

### 2.1 Simulated bus architecture & RT spoofing attack

In order to generate MIL-STD-1553 traffic which includes a specific type of RT spoofing attack, we simulated a bus architecture of a simplified fly-by-wire^1^ control system with a bus controller (BC) and several RTs, including an RT for the flight control computer, an RT for the ailerons^2^ controller and a malicious RT. Some of the RTs possess properties whose values may be set by other RTs by issuing a *set request*. The eleven blue packets in Fig. 1 demonstrate a normal set request packet sequence that is generated when RT@1 issues a request to set the value of property #0 for RT@2.

Due to the lack of authentication in MIL-STD-1553, a malicious RT can exploit such a set request by impersonating RT@1 and setting a malicious value to property #0 which will alter the state of the aircraft [4]. For example, if RT@1 is the flight control computer and RT@2 is the ailerons controller and property #0 is the required roll value, this will allow the malicious RT to tilt the plane sideways and crash it. The eleven orange packets in Fig. 1 are injected by the malicious RT in order to carry out the attack. Packets 8M-11 M are responsible for completing a valid set request with the malicious value. Then, the malicious RT replays the first seven packets (#1 M - #7 M) in order to camouflage the attack by masking the leftover packets (8–11) as a valid set request.

### 2.2 Packet to message conversion

The simulated packets were converted into MIL-STD-1553 messages [6] which are formatted as described in Fig. 2. The first column represents the message's timestamp (ms). The second column is an indication of whether or not the error bit of the status word associated with the message is set. The third column indicates if the message is a mode command message. The fourth column represents the channel associated with a message in order to support multi-channel bus architectures. The next six columns include the following fields from the message's command word: type of communication (i.e. one of BC→RT, RT→BC or RT→RT), address of the source RT, address of the sending application/sub-system on top of the source RT, address of the destination RT, address of the receiving application/sub-system on top of the destination RT, and number of included data words. The next thirty two columns hold the values of the data words included in the message. The next column contains TRUE if the message contains the data word with the malicious property value (i.e. the message alters the aircraft's behavior) or FALSE otherwise. The last column contains TRUE if at least one of the packets comprising the message are injected or FALSE otherwise. The messages in Fig. 2 correspond to the message stream that is generated in the RT spoofing attack described in Fig. 1 (black frames), from the following packet sequences: (i) 1,2 (ii) 3,4,5 (iii) 6,7,8 M,9 M, 10 M, 11 M (iv) 1 M, 2 M (v) 3 M, 4 M, 5 M (vi) 6 M,7 M, 8,9,10,11.

### 2.3 Message stream recording

On real systems, different bus architectures may correspond to different extents of vulnerability to RT spoofing attacks and may thus require a different amount of malicious messages to achieve the attacker's objective. In our bus implementation, a higher percentage of malicious messages doesn't affect the level of threat on the aircraft because even one instance of the attack, i.e. a single occurrence of the six messages from Fig. 2, would have a catastrophic effect. Nonetheless, different bus architectures may be less vulnerable, e.g. if the value that can be set to the target property is limited and only incremental changes to the state of the aircraft can be obtained by one attack instance. In such cases, many successive attack instances will be required to gradually achieve a catastrophic effect so the percentage of malicious messages will necessarily be higher. Therefore, an IDS that successfully detects malicious activity when 1.00% of the messages are malicious, but not when only 0.01% are malicious, may still be considered secure for such bus architectures even though it isn't secure for architectures like the one we implemented.

To allow researchers to evaluate IDSs for different bus architectures with different extents of vulnerability, we recorded three streams of traffic from our simulated MIL-STD-1553 bus, that contain 0.01%, 0.10%, and 1.00% malicious messages (i.e. the **next to last** column of these messages contains TRUE). The total duration corresponding to each stream is about a minute of recording, during which the attack was repeated a different number of times in order to create the different percentages. Each stream was then split into training and test streams on the first malicious message. The resulting streams were saved in CSV files in which each row corresponds to a single MIL-STD-1553 message. These streams can be found in the included “*Non-Sequential Datasets.zip*” archive.

### 2.4 Derivation of sequence datasets

As with other temporal domains in which an anomalous occurrence order of successive data records can be an important indication of malicious activity [5], MIL-STD-1553 IDSs may also benefit from analyzing the traffic as sequences [2], [4]. To allow researchers to evaluate this option, we derive a dataset of discrete data sequences [7] from each data stream as follows: (1) each message is associated with a positive integer, called the message identifier, that represents the concatenation of its values for the following fields: src_terminal_address, src_subaddress, dest_terminal_address, dst_subaddress, word_count (i.e. two different messages having identical values for these fields will be associated with the same message identifier); (2) the stream is split into sequences, each containing the identifiers of ten consecutive messages such that every incoming message results in a new sequence and consecutive sequences may differ only by the first and last message identifier. Any sequence containing at least one malicious message (i.e. that alters the aircraft's behavior) is labeled by MALICIOUS, otherwise it is labeled NORMAL. The resulting sequence datasets were saved in CSV files formatted according to Fig. 3, and are included in the “*Sequence Datasets.zip*” archive. The Java program for deriving these sequence datasets is also included in [1] so that users may derive new datasets with different sequence lengths or sequence labeling according to whether or not they contain at least one injected message. A summary of the properties of all datasets can be found in Table 2 of [2].

## Funding

This work was supported by the KAMIN program of the Israel Innovation Authority [grant numbers 57718 and 61494].

## Footnotes

1 See also https://en.wikipedia.org/wiki/Fly-by-wire.

2 See also https://en.wikipedia.org/wiki/Aileron.
